# Infective endocarditis causing acute aortic occlusion in a patient with systemic lupus erythematosus: A rare case report

**DOI:** 10.1016/j.ijscr.2025.110907

**Published:** 2025-01-20

**Authors:** James Dodd, Amirul Hakim Ahmad Bazlee, Thomas Begg, Olufemi Oshin, Bibombe Patrice Mwipatayi

**Affiliations:** a Department of Vascular Surgery, Royal Perth Hospital, Perth 6000, Australia; b University of Western Australia, School of Surgery, Perth 6000, Australia

**Keywords:** Acute limb ischaemia, sepsis, Antiphospholipid syndrome, Infective endocarditis, Septic embolus, Case report

## Abstract

**Introduction:**

We present a unique case of acute aortic occlusion secondary to infective endocarditis (IE).

**Presentation of case:**

An Aboriginal Australian woman with systemic lupus erythematosus presented with fever, confusion, tachycardia, and tachypnoea and had cold, pulseless, insensate, and paralysed lower limbs. Computed tomography angiography revealed multifocal occlusion of the distal aorta and lower limb vessels. Broad-spectrum antibiotic therapy was initiated alongside heparin infusion, and thrombectomy and four-compartment fasciotomy of the lower limbs were performed. IE, thrombotic thrombocytopenic purpura, and antiphospholipid syndrome were considered. IE was confirmed by the presence of methicillin-susceptible *Staphylococcus aureus* in blood cultures and new valvular echogenic lesions on echocardiography. Magnetic resonance imaging revealed diffused T9–T11 spinal cord infarcts. She received a prolonged course of intravenous antibiotics and intensive care and was hospitalised for 9 months. Thereafter, the patient was able to transfer and ambulate independently on flat surfaces and was discharged.

**Discussion:**

IE is associated with significant mortality and morbidity. It is commonly caused by *S. aureus*. Embolic events occur in 80 % of patients. Acute aortic occlusion secondary to IE has rarely been reported in the literature, and this is the first reported case involving the bilateral lower limbs.

**Conclusion:**

Patients may develop severe infections owing to immunosuppression. Septic emboli can occlude major arteries and cause acute limb ischaemia. A good understanding of the pathophysiology and aetiology of systemic thrombosis will lead to a thorough and broad consideration of differential diagnoses, especially for patients with complex comorbidities and a history of rheumatological disease and immunosuppression.

## Highlights

• Infective endocarditis has high mortality and morbidity rates.
• Septic emboli can occlude any artery, manifesting as acute limb ischaemia.
• Understanding systemic thrombosis pathology leads to better differential diagnoses.
• Immunosuppressed patients can develop severe manifestations of common infection.

## 1 Introduction

We present an unusual and severe case of acute aortic occlusion secondary to IE in a patient with systemic lupus erythematosus (SLE) who required surgical revascularization and prolonged complex care at a tertiary healthcare service. This case outlines the importance of a multidisciplinary approach for a life-threatening surgical presentation with a rare medical cause. This study was conducted according to the SCARE criteria [1].

## 2 Presentation of case

A female Aboriginal Australian in her 30s was brought by ambulance to a rural hospital's emergency department after being found at home an hour prior to presentation with acute-onset confusion and bilateral lower-limb paralysis.

Her medical history included SLE, class 4 lupus nephritis, and necrotising fasciitis of the hand requiring anterolateral thigh free-flap reconstruction complicated by postoperative deep vein thrombosis. The patient was receiving immunosuppressive therapy, including 1 g mycophenolate twice daily and 7.5 mg prednisolone daily. She was a nonsmoker and did not consume alcohol or use recreational drugs.

The initial examination revealed a respiratory rate of 24 breaths per minute, sinus tachycardia (165 beats per minute), body temperature of 39.0 °C, and a Glasgow Coma Scale score of 14 (E4 M4 V6). Her lower limbs were cold, pulseless, insensate, and paralysed.

### 2.1 Investigation

**Fig. 1:**
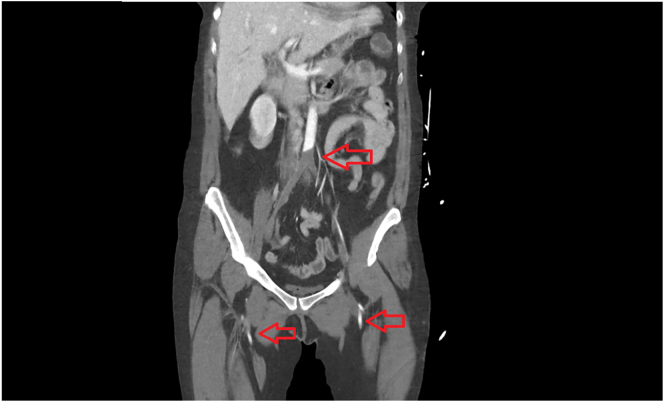
Coronal computed tomography angiography showing aortoiliac occlusion. Reconstitution of flow is evident in the bilateral superficial femoral arteries.

Initial laboratory findings indicated a haemoglobin level of 112 g/L; white blood cell count, 7.61 × 10 [9]/L; platelet count, 134 × 10 [9]/L; C-reactive protein, 8.2 mg/L; International Normalised Ratio, 1.2; activated partial thromboplastin time (aPTT), 49.3 s; fibrinogen, 1.7 g/L; and lactate, 4.8 mmol/L which is consistent with limb ischaemia and consumptive coagulopathy of sepsis. Repeat tests within 4 h revealed worsening thrombocytopaenia (93 × 10 [9]/L) and leukocytosis (19.9 × 10 [9]/L) also consistent with sepsis. Blood cultures were also performed. Computed tomography angiography revealed distal abdominal aortic occlusion extending bilaterally to the common and external iliac arteries as well as the right internal iliac artery (Fig. 1). The left profunda femoris and popliteal arteries were occluded. Low-density lesions in the left ventricle and segmental infarcts in the spleen and kidneys were visible.

### 2.2 Treatment

Owing to the suspicion of sepsis, intravenous piperacillin/tazobactam and vancomycin were administered according to local guidelines, along with a weight-based heparin infusion per protocol with a loading dose, aiming for an aPTT of 60–80 s. The patient was urgently flown to a tertiary metropolitan hospital for vascular surgery.

Upon arrival, the patient was transferred directly to the operating room. Rapid assessment confirmed the previous examination findings. Her feet were pale without fixed mottling. The patient underwent emergency aortoiliac thrombectomy and bilateral four-compartment fasciotomy, followed by angiography, 4 h post symptom onset. Aortoiliac thrombectomy was performed using a size 5 Fogarty balloon catheter (Edwards Lifesciences, Irvine, CA, USA) after bilateral common femoral arterial cutdowns. The thrombus samples were sent for microbiological analyses. The superficial and profunda femoris arteries were trawled using size 3 Fogarty balloon catheters. The final angiogram revealed aortoiliac and lower-limb arterial patency. Both feet were pink, the capillary refill time was <2 s, and palpable dorsalis pedis pulses were noted. The patient was transferred to the intensive care unit (ICU) postoperatively.

### 2.3 Outcome and follow-up

Given the presence of multiple emboli in this patient with known SLE, the rheumatology and haematology teams were consulted to confirm or rule out the presence of antiphospholipid syndrome. Thrombotic thrombocytopenic purpura was also suspected. The patient underwent plasma exchange for 5 days while awaiting the results of screening tests for antiphospholipid syndrome and other rheumatological diseases. The results were negative for antiphospholipid antibodies, including lupus anticoagulant, beta-2 glycoprotein, and anticardiolipin antibodies; each was tested twice, both with and without heparin treatment. Her A-disintegrin and metalloproteinase with thrombospondin type 1 motif, member 13 levels were 32 %.

**Fig. 2:**
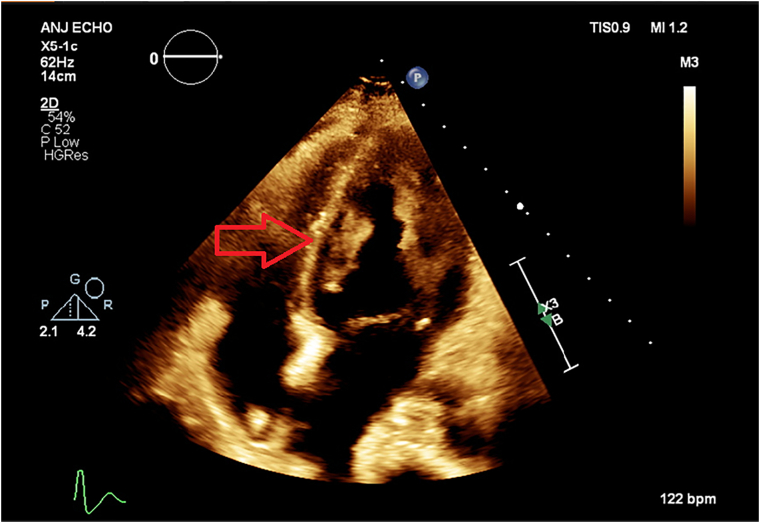
Three-chamber transthoracic echocardiography showing a mobile echogenic structure at the inferolateral wall of the left ventricle extending to the mitral valve.

**Fig. 3:**
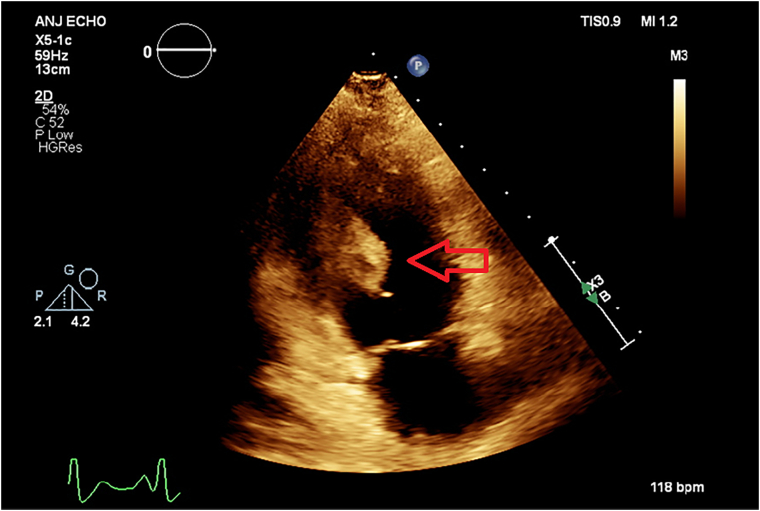
Two-chamber transthoracic echocardiography showing the thrombus.

**Fig. 4:**
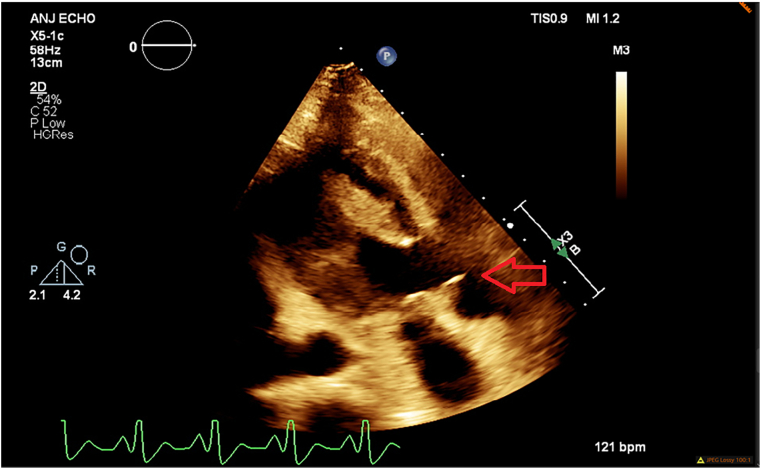
Four-chamber transthoracic echocardiography showing evidence of aortic valve nodules.

**Fig. 5:**
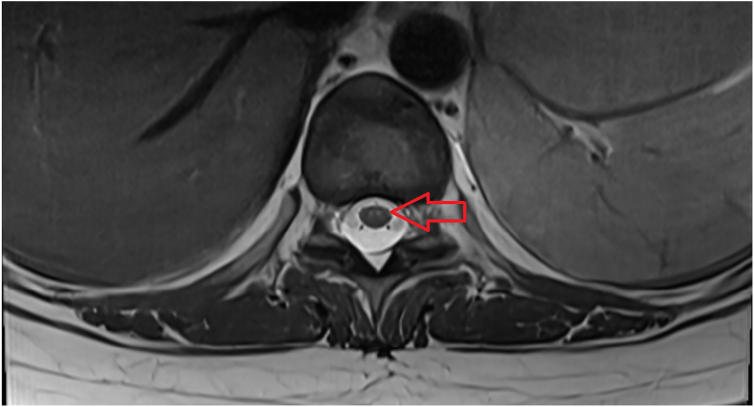
Axial T2-weighted magnetic resonance imaging of the T9–T11 intervertebral disc showing two areas of infarction.

Initial blood cultures and aortic thrombus samples revealed the presence of methicillin-susceptible *Staphylococcus aureus*. Therefore, the treatment was switched to intravenous cefazolin. Transthoracic echocardiography revealed a 4 × 2.07 × 1.5-cm mobile, echogenic structure on the inferior wall of the left ventricle extending to the mitral valve, a 0.9 × 0.7-cm echogenic structure attached to the interventricular septum of the right ventricle, and nodular echogenic lesions on the tricuspid and aortic valves (Fig. 2, Fig. 3, Fig. 4). Moderate mitral and tricuspid regurgitation were observed with severe pulmonary hypertension. After failure to regain baseline motor function, magnetic resonance imaging of the head and spine revealed small, diffuse, acute, and subacute white and gray matter infarcts as well as microhaemorrhages intracranially and between T9 and T11 (Fig. 5).

The patient was diagnosed with IE with disseminated intravascular coagulation complicated by a septic aortic thrombus and acute limb ischaemia. After consultation with cardiologists and cardiothoracic surgeons, the patient was managed non-operatively due to concerns for worsening neurological injury. Intravenous cefazolin was continued for 6 weeks.

Her condition during her ICU stay was complicated by the need for vasopressor treatment, pancytopaenia, and anuric renal failure (her creatinine level increased to 378umol/L, and her estimated glomerular filtration rate decreased to 13 mL/min), requiring temporary dialysis. After being transferred to the ward, her condition was further complicated by the development of transfusion-dependent haematomas in the right psoas and left rectus abdominis, probably due to warfarin administration, which were conservatively managed.

A reduction in the size of the left ventricular thrombus was observed prior to cessation of antibiotics. After 4 months of hospitalisation, the patient was transferred for inpatient rehabilitation for incomplete T9 paraplegia. The patient was discharged after 5 months. Fourteen months after her index event, she can mobilise short distances independently, although is reliant on her upper limbs for transfers.

## 3 Discussion

IE is an uncommon disease associated with high morbidity and mortality rates, affecting 3–7 individuals per 100,000 per year [2]. The incidence has been increasing in the past 30 years, although this has plateaued [3]. In terms of disability-adjusted life-years, >1.5 million people die each year, and the 30-day mortality rate can exceed 30% [2,4].

>80 % of IE cases are caused by gram-positive bacteria, mainly Staphylococci, Streptococci, and Enterococci. *S. aureus* is currently the most common cause of IE in developed countries, accounting for approximately 30 % of cases [5,6] and is spread because of intravenous drug use, infected healthcare workers, immunosuppression, long-term intravenous lines, and invasive procedures [5,7]. Staphylococcal IE is independently associated with mortality and the increasing prevalence of antibiotic-resistant strains, making treatment challenging [5,8]. Approximately 4 % of cases are caused by gram-negative bacterial infections, 2 % by fungal infections, and approximately 12 % are culture-negative [5,9].

Approximately 80 % of patients develop fever, 48 % develop heart murmur, and 20 % experience changes in a pre-existing heart murmur [10]. Less common signs include haematuria; arthralgia; splenomegaly; and peripheral stigmata such as splinter haemorrhages, Janeway lesions, clubbing, and retinal Roth spots [10,11]. Sepsis is an uncommon presentation of IE and is associated with a high risk of in-hospital mortality [12,13]. Embolic events occur in >80 % of patients with IE [14]. Symptomatic emboli occur in 20 %–50 % of patients and can occur in the pulmonary or systemic circulation [15,16]. The best predictor of an embolic event is the size and mobility of the vegetation, as vegetation exceeding 10 mm in length increases the risk of embolism [15]. Large (10–15 mm) staphylococcal vegetation affecting the mitral valve is associated with the highest risk of embolism [16,17]. Most septic emboli in patients with left-sided IE occur in the brain (48 %–65 %), with the musculoskeletal system and abdominal/retroperitoneal viscera commonly involved [18]. Vascular involvement is uncommon (3 %–6 %) and typically presents as mycotic aneurysms [18,19].

For patients with SLE, guidelines suggest vaccination and early recognition of infection through patient education is key to prevent poor outcomes [20]. Guidelines do not recommend prophylaxis against endocarditis.

Acute limb ischaemia caused by IE is rarely reported in the literature [21]. Aortic occlusion due to IE is exceedingly rare. Given the history of SLE, the treatment of this case was challenging. The main differential diagnosis was antiphospholipid syndrome considering that the patient was seronegative and part of an underrepresented racial minority group. Furthermore, anticoagulation can give a false negative lupus anticoagulant result which made it difficult to differentiate sepsis from catastrophic antiphospholipid syndrome.

This case contributes to the current understanding of IE and acute limb ischaemia. Furthermore, it outlines the key differential diagnoses in medically complex patients. A broad consideration of differential diagnoses is essential for the timely management of such patients.

## 4 Conclusion

This case report emphasizes the high mortality and morbidity associated with IE, which can lead to septic emboli that occlude arteries and cause acute limb ischemia. A thorough understanding of the pathophysiology of systemic thrombosis is essential for considering differential diagnoses, particularly in patients with complex comorbidities. Additionally, immunosuppressed individuals may experience more severe manifestations of infection. This case highlights the importance of a multidisciplinary approach to life-threatening arterial thrombosis.

## Credit authorship contribution statement

James Dodd: Conceptualisation, Resources, Data curation, writing – original draft, Writing – review and editing. Amirul Ahmad Bazlee: Writing – original draft, review, and editing. Thomas Begg: Resources, Data curation, Writing – original draft. Olufemi Oshin: Writing – review and editing. Bibombe Mwipatayi: Writing – review and editing, Supervision.

## Consent

Written informed consent was obtained from the patient for publication of this case report and accompanying images. A copy of the written consent is available for review by the Editor-in-Chief of this journal on request.

## Ethical approval

Case reports are exempt from requiring ethical approval in our institution as per Royal Perth Hospital Human Research Ethics Committee (Research Hub, East Metropolitan Health Service, Perth, Australia).

Case reports are exempt from requiring ethical approval in our institution.

## Guarantor

Dr. Bibombe. Patrice. Mwipatayi.

## Sources of funding

Nil

## Registration of research studies

N/A

## Declaration of competing interest

None.
